# Evaluation of the Ecotoxicity of Sediments from Yangtze River Estuary and Contribution of Priority PAHs to Ah Receptor-Mediated Activities

**DOI:** 10.1371/journal.pone.0104748

**Published:** 2014-08-11

**Authors:** Li Liu, Ling Chen, Ying Shao, Lili Zhang, Tilman Floehr, Hongxia Xiao, Yan Yan, Kathrin Eichbaum, Henner Hollert, Lingling Wu

**Affiliations:** 1 Key Laboratory of Yangtze Water environment, Ministry of Education, Tongji University, Shanghai, China; 2 Department of Ecosystem Analysis, Institute for Environmental Research (Biology V), Aachen Biology and Biotechnology, RWTH Aachen University, Aachen, Germany; 3 College of Resources and Environmental Science, Chongqing University, Chongqing, China; 4 School of Environment, Nanjing University, Nanjing, China; Indian Institute of Toxicology Reserach, India

## Abstract

In this study, in vitro bioassays were performed to assess the ecotoxicological potential of sediments from Yangtze River estuary. The cytotoxicity and aryl hydrocarbon receptor (AhR)-mediated toxicity of sediment extracts with rainbow trout (*Oncorhynchus mykiss*) liver cells were determined by neutral red retention and 7-ethoxyresorufin-*O*-deethylase assays. The cytotoxicity and AhR-mediated activity of sediments from the Yangtze River estuary ranged from low level to moderate level compared with the ecotoxicity of sediments from other river systems. However, Yangtze River releases approximately 14 times greater water discharge compared with Rhine, a major river in Europe. Thus, the absolute pollution mass transfer of Yangtze River may be detrimental to the environmental quality of estuary and East China Sea. Effect-directed analysis was applied to identify substances causing high dioxin-like activities. To identify unknown substances contributing to dioxin-like potencies of whole extracts, we fractionated crude extracts by open column chromatography. Non-polar paraffinic components (F1), weakly and moderately polar components (F2), and highly polar substances (F3) were separated from each crude extract of sediments. F2 showed the highest dioxin-like activities. Based on the results of mass balance calculation of chemical toxic equivalent concentrations (TEQs), our conclusion is that priority polycyclic aromatic hydrocarbons indicated a low portion of bio-TEQs ranging from 1% to 10% of crude extracts. Further studies should be conducted to identify unknown pollutants.

## Introduction

Marine ecosystem contamination has been greatly affected by human activities. As a coastal transitional system, an estuary serves as a recipient of environmental contaminants derived from land and rivers as well as the atmosphere. Sediments in estuaries are the main sinks of numerous potential chemical and biological pollutants. These sediments may also be considered as a secondary source of water pollution or be absorbed by benthic organisms via bioaccumulation. Thus, sediments as contaminants in rivers and estuaries may pose a potential threat to aquatic organisms and human health.

Toxicity biotests are necessary to assess sediment quality, because they can determine the effect of chemicals in sediments on organisms directly [1]. Some studies have shown many species, such as bacteria, microalgae, yeast, and fish, are widely used to assess the acute toxicity of sediments [2], [3]. To obtain comprehensive insights into potential ecotoxicological effects, the specific effects such as mutagenic, genotoxic, and dioxin-like responses should also be assessed. In vitro cell-based bioassays are of great benefit to characterize mechanism-specific activities of environmental contaminants because they are sensitive and require a smaller amount of sample than whole animal experiments [3]. Fish cell lines have been successfully used as a biological alternative of animal tests to assess the toxic effects of sediment extracts [4], [5]. For example, rainbow trout (*Oncorhynchus mykiss*) liver cell line (RTL-W1) exhibits high sensitivity to the cytotoxicity of pure substances [5], [6]; moreover, this cell line can highly express cytochrome P4501A (CYP1A)-based EROD activity upon exposure to dioxin-like compounds [1]. As such, RTL-W1 is commonly used to determine the acute cytotoxicity and CYP1A-based EROD activity of sediment extracts [2], [7].

Effect-directed analysis (EDA) is a powerful tool used to identify toxic substances in complex environmental samples [8]. This method is based on a combination of biotests, fractionation procedures, and chemical analysis. EDA can be performed to evaluate the toxic potencies of substances in sediments. This method was applied to analyze toxic chemicals in complex environmental samples. Tetrabromobisphenol A diallyl ether was identified as an emerging neurotoxicant in environmental samples by bioassay-directed fractionation and high-performance liquid chromatography-atmospheric pressure chemical ionization-tandem mass spectrometry [9]. Applying EDA to evaluate sediment extracts in Bitterfeld, Germany, where dinitropyrenes and 3-nitrobenzanthrone are quantitatively identified as the main mutagens [10]. Other studies have also successfully applied EDA to identify EROD-inducing compounds in sediments [11], [12].

Polycyclic aromatic hydrocarbons (PAHs) have been extensively investigated because these substances are widely distributed in sediments and may function as mutagens and carcinogens [13]. As dioxin-like compounds, PAHs can induce the biotransformation of CYP1A enzyme in cells by binding its ligand to aryl hydrocarbon receptor (AhR) [14]. PAH concentrations have increased as a result of the continuous and rapid development of China’s economy; these compounds have been detected in the sediments of coastal embayment, continental shelf in China, and Yangtze River estuary [15], [16].

The Yangtze River estuary, one of the largest estuaries in the world, is located in the east of China and adjacent to the East China Sea. With agricultural and industrial developments in this region, numerous pollutants have been discharged into this estuary [17]. The increasing fraction of wastewater in the Yangtze River increases the levels of nutrients, heavy metals, and dissolved organic carbon; in addition to wastewater, industrial organic chemicals at concentrations of 500 kg to 3,500 kg per day [18]. Approximately 206 hazardous organic chemicals have been detected in water, and 106 chemicals have been found in sediments; among these chemicals, 17 are listed as priority controlled pollutants in America [19]. Studies have also indicated that various organic contaminants, such as PAHs [16], polychlorinated dibenzo-*p*-dioxins (PCDDs)/polychlorinated dibenzofurans (PCDFs) [20], and polychlorinated biphenyls (PCBs) [21], are present in sediments from the Yangtze River estuary. In addition, newly emerging contaminants, such as polybrominated diphenyl ethers and perfluorinated compounds, have been extensively investigated because of their presence in the Yangtze River [22], [23]. Despite the polluted state of this river, studies have been rarely conducted regarding the ecotoxicological potential of Yangtze River sediments [17], [24].

In this study, RTL-W1 cells were used to determine the acute cytotoxicity and CYP1A-based EROD activity of the sediment extracts from the Yangtze River estuary. EDA was applied to identify the potential hazardous substances causing AhR-mediated activities. PAHs were chemically analyzed to obtain comprehensive information on these sediments and estimate the extent to which these sediment extract chemicals elicit EROD-inducing effects. The main objectives of this study are listed as follows: (1) to assess the ecotoxicological potential of sediments from the Yangtze River estuary by evaluating the cytotoxic and AhR-mediated effects on RTL-W1 cells; (2) to identify the sediment fractions causing such AhR-mediated effects; and (3) to determine the contribution of priority PAHs to EROD induction of sediment samples.

## Materials and Methods

### 2.1 Sampling

**Figure 1:**
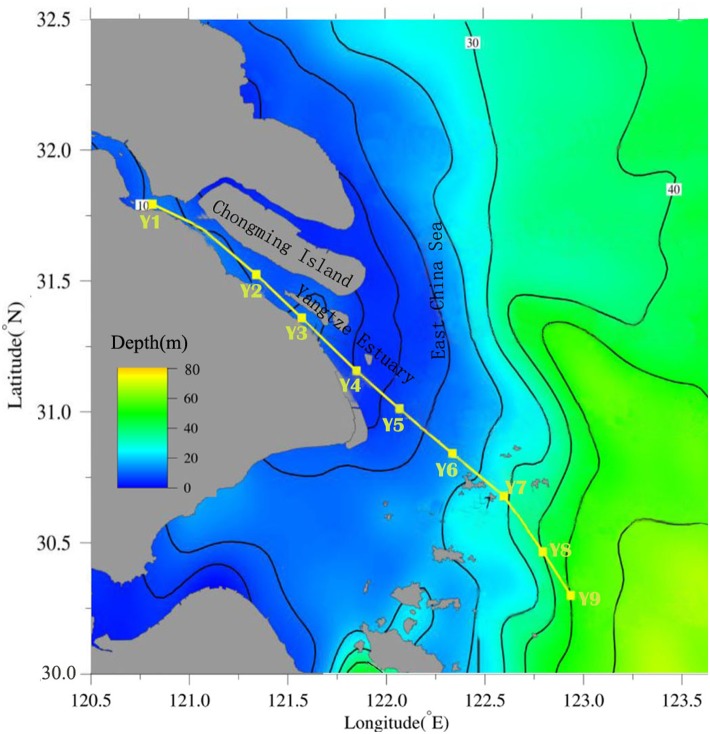
Map of the sampling locations in the Yangtze River estuary. Nine samples Y1–Y9 were collected along the estuary in March 2012.

No specific permissions were required for the completion of this study as the field measurements did not involve endangered or protected species nor were conducted in a specified protected area. The surface layer of sediment samples (0 cm to 5 cm) were collected from nine locations in the Yangtze River estuary in March 2012. For each sampling locations, four samples was collected and then mixed to form one sample. The sampling locations are shown in Figure 1. Samples were collected along the salinity gradient and location information is presented in Table S1. Samples Y1 to Y3 were fresh water dominated sediments. Sites Y4 and Y5 were located in the turbidity maximum zone and the samples were brackish water dominated sediments. Sites Y6 to Y9 were located in the river plume zone and the samples were marine sediments. Sediments were collected from each location by using a stainless steel grab sampler. Samples were shock-frozen at –20°C immediately until further processing. Afterward, these samples were freeze-dried at –50°C and sieved using a 100-mesh sieve. The samples were then stored in combusted glass with Teflon lined lids at –20°C in the dark until extraction.

### 2.2 Sediment extraction procedure

The samples were prepared according to previously described methods [25]. The freeze-dried sediment samples (20 g) were separately extracted with acetone (Merck, Darmstadt, Germany, HPLC) for 48 h by using standard reflux (Soxhlet) extractors with six cycles per hour. Elemental sulfur was removed by treating the extracts with activated copper. The extracts were then reduced in volume to obtain a final volume of 2 mL by rotary evaporation. The solvent was divided into two aliquots and concentrated close to dryness with gentle nitrogen stream. One aliquot was replaced with 1 mL of dimethylsulfoxide (DMSO; Sigma-Aldrich Chemie GmbH, Steinheim, Germany) and used for in vitro biotests, resulting in a final concentration of 10 g of sediment dry weight per mL of DMSO (10 g/mL). The other aliquot was dissolved in *n*-hexane (Merck, HPLC) and used for multilayer fractionation. The extracts were then stored at –20°C until further analysis.

### 2.3 Multilayer fractionation

To identify unknown substances that contribute to the dioxin-like potencies of whole extracts, we selected the sediments used for fractionation on the basis of maximum AhR agonist activities in crude extracts. Fractionation was performed according to previously described methods with slight modification [26]. Fractionation was also performed using a silica gel/aluminum oxide column. The column was prepared according to the following procedures. Silica gel (60 mesh to 200 mesh, Merck, Darmstadt, Germany) and aluminum oxide (50 mesh to 200 mesh, Merck, Darmstadt, Germany) were activated at 180°C for 24 h. Sodium sulfate was baked at 450°C for 5 h before use to eliminate possible production residues, such as phthalates. Glass columns (inner diameter = 10 mm and height = 50 cm) were filled from the bottom with 10 g of silica gel, 5 g of aluminum oxide, and 2 g of sodium sulfate. The gels were then washed twice with *n*-hexane. Afterward, the extracts were placed in the columns and eluted with different solvents at increasing polarities: F1 containing non-polar paraffinic components were eluted with 50 mL of *n*-hexane; F2 characterized by weakly and moderately polar components were eluted with 70 mL of *n*-hexane/dichloromethane (Merck, HPLC; 7∶3, v/v); and F3 containing highly polar components were eluted with 50 mL of acetone/methanol (Merck, HPLC; 1∶1, v/v). Using a rotary evaporator, we decreased the volumes of the eluates to 2 mL and evaporated close to dryness with gentle nitrogen stream. Afterward, the eluates were replaced with 1 mL of DMSO. These fractions were then stored at –20°C in the dark until analysis.

### 2.4 Cell culture

Cytotoxicity was assessed using the fibroblast-like permanent cell line RTL-W1 isolated from the liver of a female *O. mykiss* [6]. The cells were kindly provided by Drs. Niels C. Bols and Lucy Lee (University of Waterloo, Canada) and cultured according to the method described by Klee et al. [27].

### 2.5 Neutral red retention (NR) assay

The acute cytotoxicity of sediment extracts on RTL-W1 cells were assessed by NR assay according to Borenfreund and Puerner [28] with slight modifications by Klee et al. [27]. Sediment extracts were serially diluted with L15 medium to obtain a concentration range of 1.57 mg to 100 mg of dry sediment per mL medium with six internal replicates. 3,5-Dichlorophenol (Sigma, Seelze, Germany) was used as a positive control sample at a concentration of 80 mg/L in each test plate. All of the experiments were performed in independent triplicates. The cytotoxic potentials of individual extracts that induced 50% mortality after 48 h (48 h NR_50_) were calculated accordingly. The unit of NR_50_ was designated as mg dry weight sediment equivalent (SEQ) per mL test medium.

### 2.6 EROD induction assay

The dioxin-like activity of sediment extracts was determined by EROD induction assay according to previously described methods [29]. The highest test concentration of the EROD assay to determine the enzyme activity and avoid the cytotoxic effects based on NR_80_ obtained from a preliminary NR assay with RTL-W1 cells. The cells were seeded into 96-well microtiter plates (TPP, Trasadingen, Switzerland) and exposed to sediment extracts in eight dilution steps with six internal replicates. 2,3,7,8-Tetrachlorodibenzo-*p*-dioxin (TCDD, Promochem, Wesel, Germany) was serially diluted to obtain a final concentration ranging from 3.13 pM to 100 pM in two separate rows of each plate as a series of positive control sample. After the samples were incubated for 72 h, induction was terminated by removing the growth medium and freezing at –70°C to lyse the cells. The deethylation of exogenous 7-ethoxyresorufin was initiated by adding 7-ethoxyresorufin to each well; the resulting reaction mixture was then incubated in the dark at room temperature for 10 min before NADPH (Sigma) in PBS (Sigma) was added. The plates were further incubated for 10 min and the reaction was terminated by adding fluorescamine (Sigma) dissolved in acetonitrile (Merck, HPLC). EROD activity was determined fluorometrically after another 15 min at excitation and emission wavelengths of 544 and 590 nm, respectively, by using an Infinite M200 plate reader (Tecan, Crailsheim, Germany). The amount of protein was fluorometrically determined using the fluorescamine method at excitation and emission wavelengths of 360 and 465 nm, respectively [30]. The concentration-response curves of EROD induction in the RTL-W1 bioassay were designed by non-linear regression (Prism 4.0, GraphPad, San Diego, USA) using the classic sigmoid curve or Boltzmann curve as model equations.

AhR agonist activities were determined using a fixed-level approach [31]. Extract EC_25TCDD_ of each sediment extract was obtained and normalized to that of the positive control 2,3,7,8-TCDD as biological toxic equivalent concentrations (bio-TEQs) [29]. EC_25TCDD_ was used to compare the samples and calculate TCDD-equivalent concentrations in the samples. Bio-TEQ concentrations were calculated as follows:
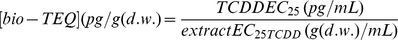
(1)where TCDD EC_25_ (pg/mL) is the concentration of the TCDD positive control sample causing 25% of EROD induction and extract EC_25TCDD_ (g/mL) is the concentration of the sediment extract equivalent causing 25% of EROD induction. EC_25TCDD_ was considered as a more appropriate measure than EC_50_ in this study because EC_50_ has not been well defined by dose-response curve in several cases [31].

### 2.7 Chemical analysis

A total of 16 priority PAHs in the crude sediment extracts described by Environmental Protection Agency (EPA) were quantified by Agilent 6890B gas chromatograph coupled to a mass selective Agilent 5977A MSD detector, which was operated in the selective ion monitoring mode. The sum parameter was calculated on the basis of 16 priority PAHs as follows: naphthalene; acenaphthylene; acenaphthene; fluorene; phenanthrene; anthracene; fluoranthene; pyrene; benzo[a]anthracene; chrysene; benzo[b,j]fluoranthene; benzo[k]fluoranthene; benzo[a]pyrene; dibenzo[a,h]anthracene; benzo[g,h,i]perylene; and indeno[1,2,3-cd]pyrene. 2-Fluorobiphenyl and terphenyl-d14 were used as surrogate standards and added before Soxhlet extraction for each sample. The detector was equipped with 30 m×0.25 µm film HP-5MS fused silica capillary column (Agilent Technologies), and 1 µL of each fraction was injected in a splitless mode. The initial temperature was set at 45°C for 2 min and then increased to 265, 285, and 320°C at a rate of 20, 6, and 10°C/min. Afterward, temperature was held constant at 320°C for 4 min. Instrumental analysis was conducted after internal standards (naphthalene-d8, acenaphthene-d10, phenanthrened10, chrysene-d12, and perylene-d12) were added.

During the analytical procedure, a procedural blank, a spiked blank, and a duplicate sample were processed. Targets were not detected in procedural blanks. The surrogate recoveries of 2-fluorobiphenyl and terphenyl-d14 were 88% (±5%) and 102% (±4%), respectively. The recoveries of targets ranged from 88% to 103% in spiked blank samples. The reported concentrations were not corrected by surrogate standard recoveries.

### 2.8 Chem-TEQ calculation

To determine the contribution of EPA-PAHs to the overall AhR-mediated activity, we determined chem-TEQs based on PAH potencies relative to 2,3,7,8-TCDD [32]. Chem-TEQs were calculated by multiplying the concentration of each AhR-active chemical by specific relative potency (REP) of RTL-W1 cells (Eq. 2) [29], [32]. Chem-TEQ concentrations at picogram PAH per gram of SEQ were calculated as picogram per gram (pg/g).
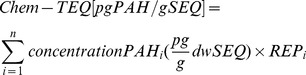
(2)


### 2.9 Data analysis

Data were expressed as mean ± SD. Statistical analyses were performed using SPSS 17.0 (SPSS lnc., Chicago, IL, USA). Maximum concentrations were used to assess the worst pollution scenario at each section compared with other sediment systems.

## Results and Discussion

### 3.1 Cytotoxicity of crude extracts and multilayer fractions

**Figure 2:**
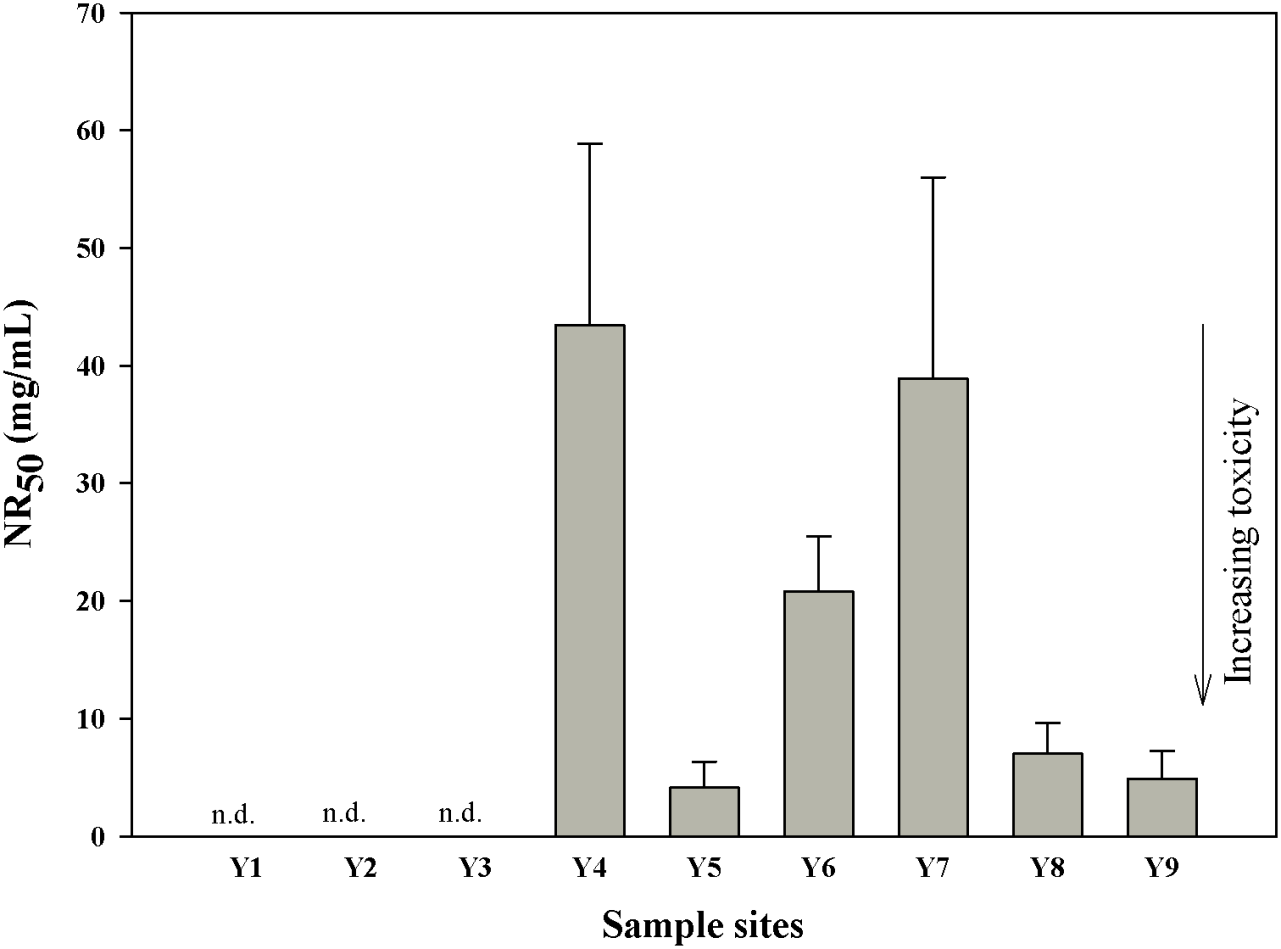
Cytotoxicity of sediment extracts from the Yangtze River estuary. n.d. = not detectable, indicating that no toxic effect of sediment extracts at a concentration of 100 mg/mL with RTL-W1 cells. NR_50_ values for sediment extracts are given in mg sediment equivalent per mL medium (mg/mL). All the values are expressed as means ± SD.

The results of the NR assay on the crude extracts are shown in Figure 2. The sediment extracts from the study sites Y1, Y2, and Y3 showed very low potency and failed to elicit cytotoxic effects at a concentration of 100 mg/mL. By contrast, extracts from the six other sites revealed evident cytotoxicity to RTL-W1 cells with NR_50_ ranging from 4.1 mg/mL to 43.4 mg/mL (Table S2). The extracts from sites Y5, Y8, and Y9 revealed relatively greater cytotoxicity than those from other sites. The lowest cytotoxic potency was obtained from extracts collected from sites Y4, Y6, and Y7. The cytotoxicity of the sediment extracts from downstream regions generally showed higher activities than those from upstream regions.

In the multilayer fractionations, only fraction F1 from sites Y5 and Y9 elicited cytotoxic effects with NR_50_ of 63.2 and 60.4 mg/mL, respectively. No cytotoxic potential was detected in F2 and F3.

According to a sediment classification system of sediments found in Germany [33], the threshold values of NR biotest system are as follows: NR_50_>80 mg/mL, non-toxic; 80≥NR_50_≥31 mg/mL, moderately toxic; and NR_50_<31 mg/mL, strongly toxic. On the basis of this threshold values, we classified the crude sediment extracts from sites Y4 and Y7 as moderately toxic and those from sites Y5, Y6, Y8, and Y9 were strongly toxic. The cytotoxicity effects elicited by the sediment extracts in this study were compared with those observed in sediment extracts from other Asian, European, and South American locations (Table 1). The cytotoxic potential of the sediment extracts from the Yangtze River estuary was comparable to that of the suspended particulate matter (SPM) and sediment extracts from the upper Danube River and the surface sediments and sediment core extracts from the upper Rhine River [5] and the Mecklenburg Bight (Western Baltic Sea) [2]. Furthermore, the cytotoxic potential of these extracts from the Yangtze River estuary was higher than that of the sediment extracts from Tietê River [34].

**Table 1:** Cytotoxicity of sediment extracts of the Yangtze River estuary and that of other sediment extracts from previous studies.

Sampling Locations	NR_50_ (mg/ml)	References
Mecklenburg Bight (Western Baltic Sea)	≥13.5	[3]
Upper Rhine (Germany)	≤50	[6]
Upper Danube River (Germany)	<40	[6]
Tietê River (Sao Paulo, Brazil)	29∼225	[35]
Yangtze River estuary (China)	4.1∼43.4	In this study

The cytotoxic effect of the sediment extracts may be attributed to the organic contaminants previously polluting the Yangtze River estuary with the rapid development of industrialization. In 2006, more than 10,000 chemical enterprises, or equivalent to approximately half of the total number of enterprises in China, were situated by the river [35]. Many petroleum and chemical plants and docks were also located along the river. In this study, high PAH concentrations, pharmaceuticals, and other organic compounds have been frequently detected in the study region [36], [37], [38]. Organic compounds, such as PAHs with two or three rings (naphthalene, acenaphthylene, acenaphthene, fluorene and phenanthrene), can elicit cytotoxic effects on rainbow trout (gill) cells [39]. Pharmaceuticals, such as substituted phenols, are also potential inducers of cytotoxicity [40]. In this study, toxic compounds may have accumulated in the Yangtze River estuary and may be accounted for the cytotoxic potential of sediment extracts.

### 3.2 AhR-mediated activities in crude sediment extracts

**Figure 3:**
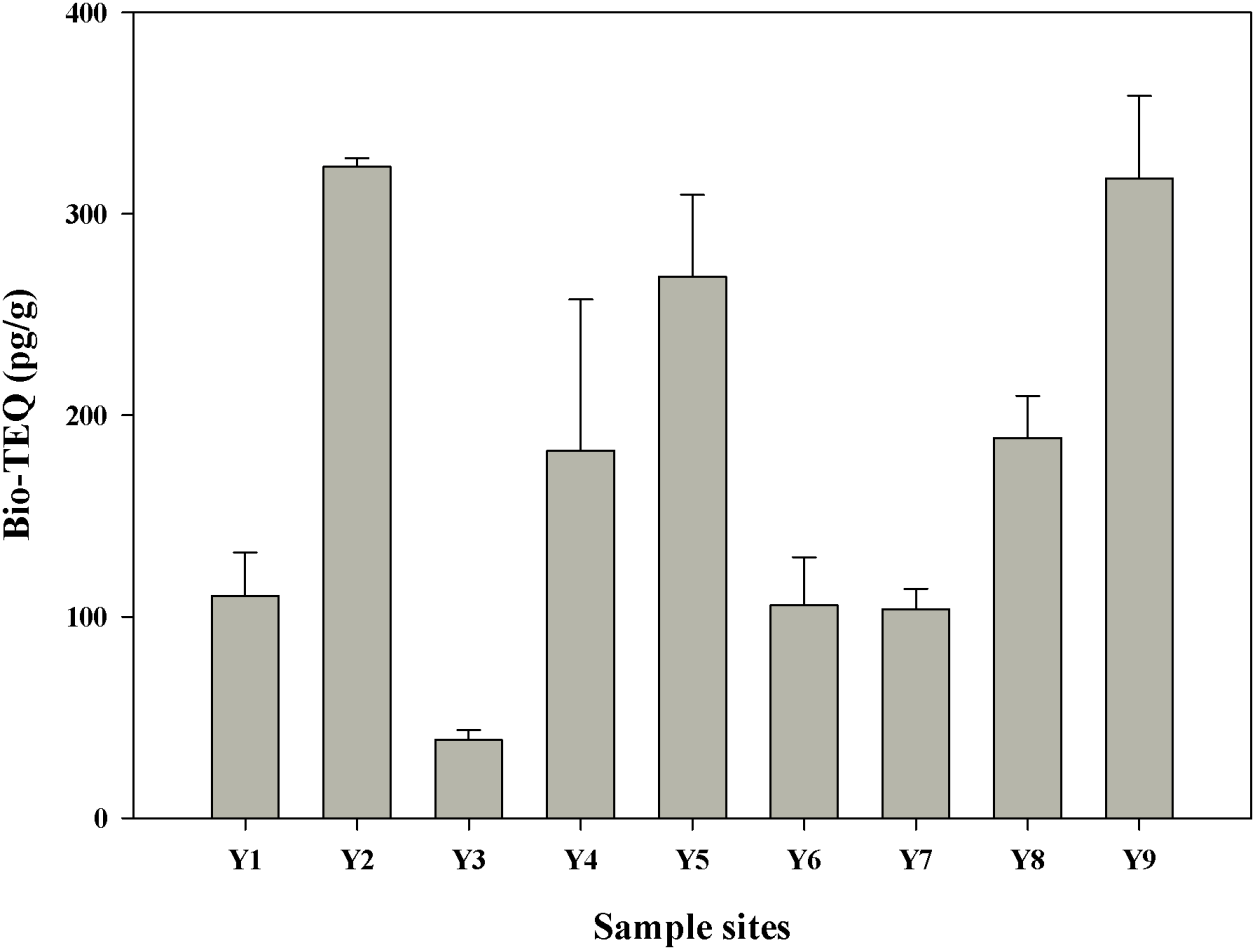
AhR-mediated activity of sediment extracts from the Yangtze River estuary. The values as determined by the EROD assay with RTL-W1 cells are expressed as biological toxicity equivalents (Bio-TEQ; pg/g dw). Data are given as means of 3 replicates ± SD.

Figure 3 shows the results of the EROD assay of the AhR agonists in the crude sediment extracts. The dioxin-like potentials of the sediment extracts among samples varied. In particular, bio-TEQ of crude extracts ranged from 38.9 pg/g dw to 323.5 pg/g dw (Table S2). A relatively high EROD induction was observed in sediment extracts from sites Y2, Y5, and Y9. By contrast, bio-TEQs of the sediments from Y3 showed relatively low EROD induction.

We obtained low to moderate values (Table 2) and corresponding AhR-mediated activity of the investigated sediments compared with bio-TEQs from other river systems. The bio-TEQs of the sediments from different locations were compared on the basis of the reported results regardless of the difference between the extraction methods. EROD induction potential of the sediment extracts from the Yangtze River estuary was comparable to that of the sediment extracts from River Elbe estuary [41] and Saginaw Bay [42]. The sediment extracts from the Yangtze River estuary showed higher EROD induction potential than those from Masan Bay [43], Tai Lake [44], and Yellow Sea [45]. The EROD induction potential of the sediment extracts from the Yangtze River estuary was also lower than that of the sediments from Tietê River [34], Morava River [14], Dagu Rivers, and Haihe [46]. Keiter et al. [33] developed a classification system for AhR-mediated toxicity of German river systems. According to this evaluation scheme, the sediment samples of the Yangtze River estuary revealed only minor AhR-mediated effects. The concentrations of organic pollutants may be lower in Yangtze River than in Rhine River; however, a comparably higher mass transport of water and particulate matter in the Yangtze River system contributes to relatively larger amounts of organic pollutants in the receiving water body of Yangtze River [17]. The mean water discharge (30,200 m^3^/s) (http://china.org.cn/english/eng-shuzi2003/gq/dili5.htm) Yangtze River is approximately 14 times greater than that of Rhine River (2,200 m^3^/s) [47]. As such, the total mass transfer of AhR compounds has been extensively observed in the China Sea because Yangtze River releases a higher discharge than European waters.

**Table 2:** AhR-mediated activity of sediment extracts of the Yangtze River estuary and those of other rivers described in previous studies.

Sampling Locations	Bio-TEQ (pg/g)	References
Masan Bay (Korea)	17∼275	[44]
River Elbe Estuary (Germany)	15.5∼322	[42]
Tietê River (Brazil)	n.d.∼24170	[35]
Saginaw Bay (USA)	11∼348	[43]
Morava River (Czech Republic)	1∼17000	[15]
Tai Lake (China)	n.d.∼114.5	[45]
Dagu Rivers (China)	1200∼13900	[47]
Haihe (China)	330∼930	[47]
Yellow Sea (China)	3.4∼28	[46]
Yangtze River estuary (China)	38.9∼323.5	In this study

Note: n.d. = not detectable or below the detection limit.

NR assay results showed that the Yangtze River estuary is polluted by a large variety of chemicals. A high concentration of organic pollutants, such as PAHs [48], PCBs, and PCDDs/PCDFs [49], which are potential inducers of the cytochrome P450 system, were detected in the sediments collected from Yangtze River estuary. These sediment-bound contaminants may affect the AhR activity of the samples. Discharges from the industries along the Yangtze River estuary should be dealt with, and measures should be established to reduce the input of toxic effluents into the estuary [18].

### 3.3 AhR-mediated activities in multilayer fractions

**Figure 4:**
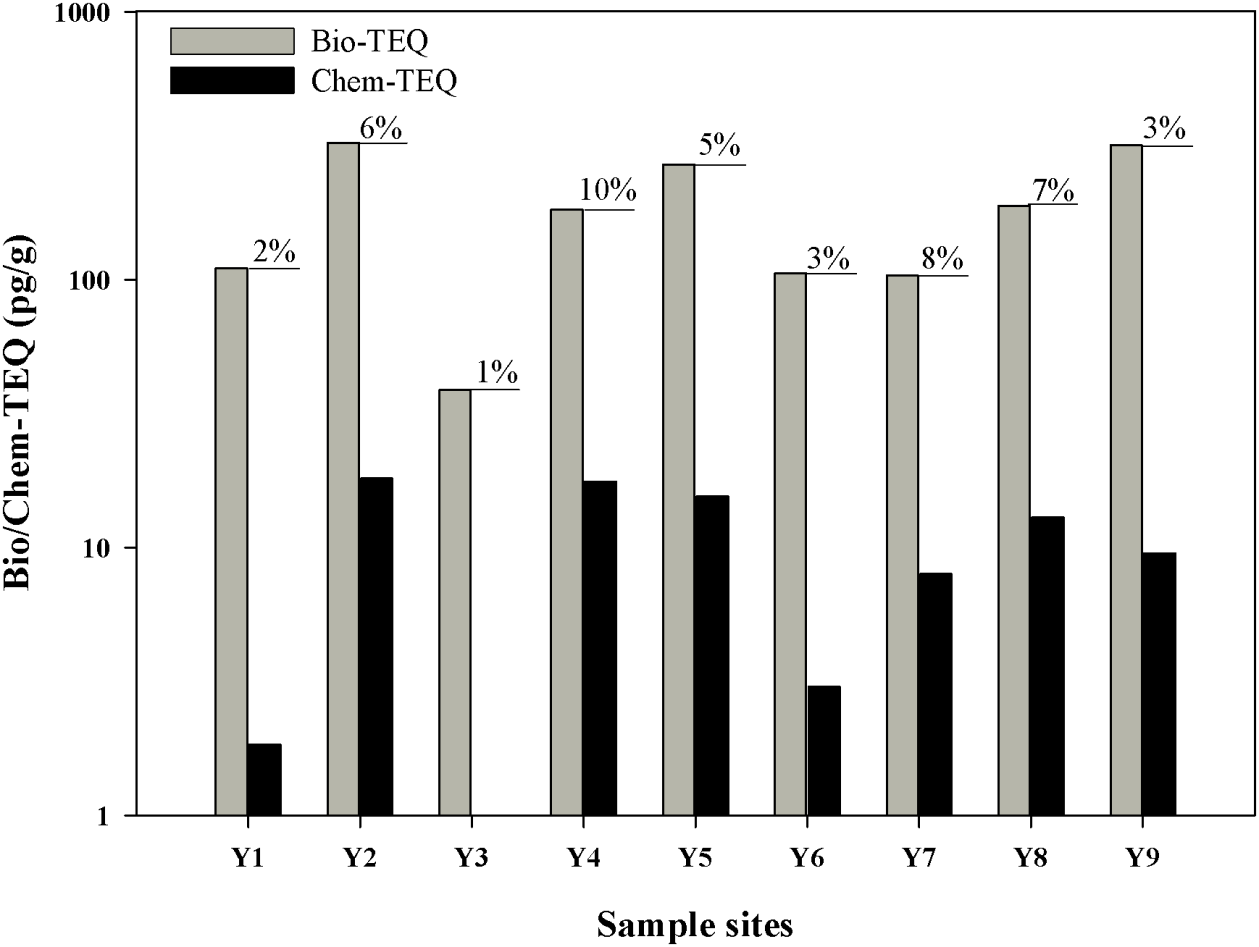
AhR-mediated activities of crude and multilayer fractions of sediment extracts from the Yangtze River estuary. The results are based on EROD inductions with RTL-W1 cells and presented as bio-TEQs. F1 contains non-polar paraffinic components. F2 contains weakly and moderately polar components. F3 contains highly polar components. The numbers in percentage were calculated as (F1+F2+F3–CE)/CE×100%. The difference between the combined F1 to F3 and the induced crude extracts induction is given. Bio-TEQs are given as means of *n* = 3 independent experiments. CE, crude extracts.

Based on EROD induction assay results of the crude extracts, the sediment samples from sites Y2, Y4, Y5, Y7, Y8, and Y9, which had the highest bio-TEQ values, were selected for further identification of AhR-mediated activities using multilayer fractionation. As shown in Figure 4, AhR-mediated activities caused by multilayer fractions (F1 to F3) were compared with crude sediment extract inductions.

Fractions F1, which contained non-polar paraffinic components, showed no or very low AhR-mediated activities. Primary fractions F2, which contained weakly and moderately polar components, showed the highest AhR-mediated activities. In addition, fractions F3, which contained more polar components, also showed significantly increased activities. The results were consistent with those of other studies performed previously. Some non-priority PAHs and more polar compounds, such as heterocyclic compounds containing nitrogen, sulfur, or oxygen heteroatoms (NSO-Het), also show dioxin-like activity [50]. The highest AhR agonistic effect of the SPM collected during a flood event from the rivers Neckar and Rhine was found in fractions containing high molecular weight PAHs with more than 16 aromatic C-atoms [51]. Engwall et al. [52] indicated that the polyaromatic fractions of the bottom sediment and suspended particulate matter showed relatively higher dioxin-like potencies than the other fractions. Therefore, weakly and moderately polar components and more polar components are major inducers of AhR-mediated activities and require further study.

However, AhR-mediated activities showed site-specific differences among locations (Figure 4). Sample sites Y4, Y7, and Y8 (especially site Y4) showed significantly greater bio-TEQs for the sum of primary fractions F1 to F3 than the crude extracts, and the percentages of the exceeded parts were 62%, 40%, and 32%, respectively (Table S3). Sample site Y5 exhibited comparable bio-TEQs of crude extracts to the added primary fractions.

Interactions, such as synergistic or antagonistic effects, exist in different substances and may lead to changes in the effects of certain substances. Low EROD activities do not necessarily indicate low induction potency but may have been caused by high concentrations of inducers and non-inducers that inhibit EROD activity [53]. In crude extracts, the low bio-TEQs might be explained by EROD antagonistic and inhibiting effects because sediments contain a broad range of unspecified compounds [2]. EROD inductions with more than 100% induction in fractions compared with crude extracts may be caused by the retention of humic substances during the fractionation procedure [54]. The separation of aliphatic and polar compounds from nonpolar aromatics resulted in an activity of a fraction that exceeds that of the crude sediment extract in the Neckar River basin [11]. Thus, in this study, some antagonistically acting substances were probably separated in the process of elution, and thus, agonists could completely display activity.

In contrast, sites Y2 and Y9 showed decreased induction when comparing bio-TEQs of F1 to F3 with the crude extract. This phenomenon can be explained as follows: fractionation of mixtures of bioactive compounds into multiple fractions may have reduced the individual bioactivity to below detection limits, thereby efficiently rendering some of the active compounds non-detectable by the bioassay [55]. Thomas et al. [56] applied the EDA method to analyze the estrogen receptor agonist of water extracts. After fractionation, the sum of activity of the individual fractions was less than 20% of the activity of the crude extract. The crude extracts are a complex mixture of unknown substances, and thus, the full recovery of all effect compounds within the samples was difficult. The AhR-mediated activities of the sediment extract fractions are no longer observed in the present study, indicating that the reduced complexity of the mixture also reduces toxicity.

### 3.4 Concentrations of PAHs

Chemical analysis was applied to determine the concentrations of the 16 EPA-PAHs listed in Table 3. Most of the 16 EPA-PAHs were detected in all the sediment samples from the Yangtze River estuary. The highest concentrations of total PAHs were found in site Y2, and the lowest concentrations were found in site Y1 and Y3. The concentrations of total PAHs ranged from 21.5 ng/g dw to 190.5 ng/g dw sediment. Some studies reported the levels of PAHs in the sediment from different sections of the Yangtze River. Concentrations of total PAHs at the Chongqing section was 257 ng/g dw to 723 ng/g dw, and at Jialing River, a tributary of the upper Yangtze River, was 132 ng/g dw to 349 ng/g dw [57]. Concentration of PAHs in the Wuhan section was high, ranging from 303 ng/g to 3,995 ng/g in the main stream and 4,121 ng/g to 4,262 ng/g in the tributaries [58]. Compared with other sections of the Yangtze River, the concentration of PAHs in sediment from the Yangtze River estuary was relatively low [17]. The dilution from the East China Sea may be one of the reasons for such low concentration [59]. In addition, Wang et al. [60] showed that the PAH composition in the Yangtze River estuary, which was dominated by four-ring to six-ring PAHs, was mainly caused by petroleum combustion, vehicle emission, and biomass combustion (mainly coal) in the nearshore area, whereas PAHs composition, which was dominated by two-ring to three-ring PAHs, in the farther shore zone originated from petroleum combustion of shipping processes and shoreside discharges.

**Table 3:** Concentrations of the 16 US EPA-polycyclic aromatic hydrocarbons (PAHs; ng/g dw) in sediment samples from the Yangtze River estuary.

Sampling site	Y1	Y2	Y3	Y4	Y5	Y6	Y7	Y8	Y9
Naphthalene	6.0	47.0	11.0	22.0	7.0	4.0	26.0	7.0	8.0
Acenaphthylene	n.d.	4.0	n.d.	1.0	2.0	1.0	2.0	2.0	2.0
Acenaphthene	n.d.	2.0	n.d.	2.0	2.0	n.d.	n.d.	1.0	2.0
Fluorene	1.0	5.0	1.0	3.0	6.0	2.0	3.0	4.0	4.0
Phenanthrene	2.0	15.0	1.0	14.0	12.0	5.0	6.0	11.0	9.0
Anthracene	2.0	4.0	1.0	3.0	4.0	1.0	2.0	3.0	2.0
Fluoranthene	2.0	17.0	1.0	20.0	13.0	4.0	6.0	10.0	7.0
Pyrene	1.0	8.5	0.5	10.0	7.0	2.5	3.0	5.5	4.5
Benzo[a]anthracene	1.0	13.0	1.0	14.0	10.0	3.0	5.0	8.0	6.0
Chrysene	2.0	14.0	1.0	13.0	11.0	3.0	5.0	9.0	6.0
Benzo[b]fluoranthene	2.0	19.0	1.0	16.0	18.0	6.0	9.0	14.0	12.0
Benzo[k]fluoranthene	1.0	5.0	n.d.	6.0	5.0	1.0	3.0	4.0	3.0
Benzo[a]pyrene	n.d.	14.0	1.0	13.0	10.0	n.d.	4.0	9.0	6.0
Indeno[1,2,3-cd]pyrene	1.0	10.0	1.0	9.0	8.0	2.0	4.0	7.0	5.0
Dibenz[a,h]anthracene	n.d.	3.0	n.d.	2.0	2.0	n.d.	1.0	2.0	1.0
Benzo[g,h,i]perylene	1.0	10.0	1.0	8.0	8.0	2.0	4.0	7.0	5.0
**Sum of EPA-PAHs**	**22.0**	**190.5**	**21.5**	**156.0**	**125.0**	**36.5**	**83.0**	**103.5**	**82.5**

Note: n.d. = not detectable or below the detection limit.

### 3.5 Correlation between bio-TEQs and chemical analyses

**Figure 5:**
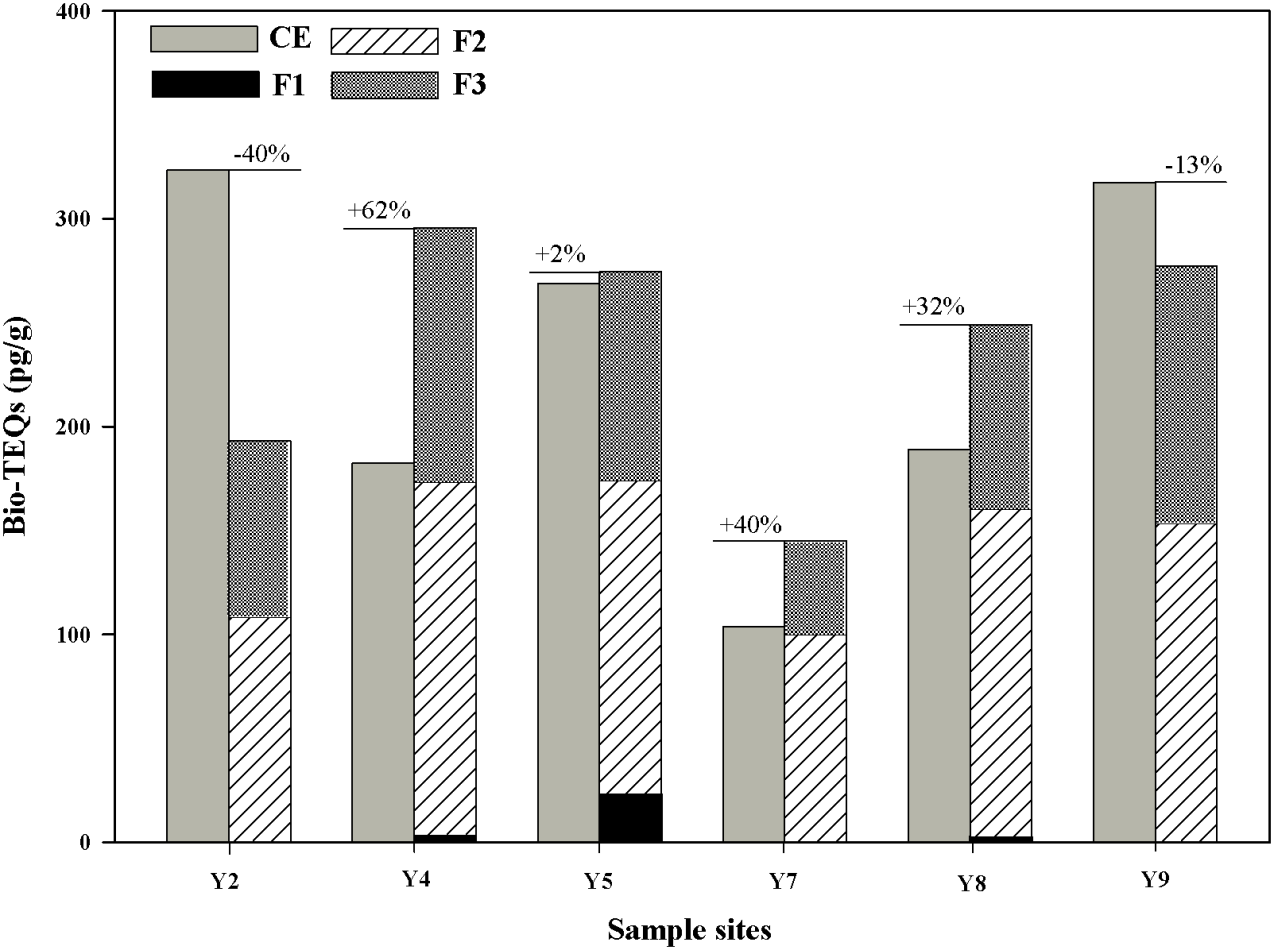
The contribution of Chem-TEQs of PAHs to Bio-TEQs. The total biological response of the crude extracts in the EROD assay with RTL-W1 cells are expressed as bio-TEQs. The chem-TEQs of the 16 measured EPA PAHs were calculated by multiplying compounds concentrations and relative equivalency potencies and are given in pg/g dw. Numbers in percent indicated the calculated contribution of these PAHs to the EROD induction in each crude extract.

According to Bols et al. [32], chem-TEQs were calculated by multiplying the specific REP factors for RTL-W1 cells with corresponding compound concentrations. The total biological response in the EROD assay (Bio-TEQs) and chem-TEQs of PAHs are shown in Figure 5. For sites Y1, Y3, Y6, and Y9, less than 5% of the induction could be explained by known priority PAHs, which were expressed as chem-TEQs. For sediment extracts from sites Y4 and Y7, 10% and 8% of the induction could be attributed to the presence of analyzed PAHs, respectively.

Several studies have shown that halogenated aromatic hydrocarbons and PAHs are chemicals that are usually related to dioxin-like activity, and can act as AhR agonists [11], [61]. Generally, chemical analysis focused on substances that were considered to be a priority or are more relevant, and other non-priority substances were ignored. However, bio-TEQs are not explained by the chem-TEQs calculated from the concentrations of the analyzed priority compounds. In this study, less than 10% of the bio-TEQs could be attributed to the EPA-PAHs, whereas non-priority substances were shown as high inducers.

Concentrations of pollutants at different matrixes were based on reported results, and differences between the analytical methods have been neglected. According to Keiter et al. [7], 55% to 88% of AhR-mediated responses observed in upper Danube River sediments were not due to the measured priority PAHs, PCBs, and PCDDs/PCDFs. The highest bio-TEQ of sediments core layers (2.26×10^5^ pg/g) was sampled at the historically contaminated dumping site in the Baltic Sea. This value was much higher than that obtained in this study, but it was lower than expected from the PAH concentration in the samples from the dumping site [2]. Special interactions of some contaminants or of contaminants with sediment components might be responsible for such low value. In contrast, the majority of bio-TEQ (approximately 58%) measured from the extracts of sediments from the Elbe River by EROD assays could be due to the priority PAHs [41]. In a number of studies involving sediments collected from the Elbe, Tietê, and Danube Rivers, the chemical measurements of priority PAHs or other persistent organic pollutants, such as PCBs and PCDDs/PCDFs, could only account for a small portion of the AhR-mediated potency [7], [11], [34]. The results from these above mentioned studies were consistent with those obtained in the present study. Kaisarevic et al. [12] showed that only minor portions of biologically derived TCDD-TEQs from waste water canal sediments could be due to the monitored PAHs with known relative potencies. In a previous study, PCBs and PCDDs/PCDFs were responsible for a minor portion of the total AhR-mediated activities of SPM in two floods [29]. The non-priority pollutants mainly mediated the high induction rates. Therefore, the main induction in the biotest systems was caused by non-priority pollutants [11], [62]. Some studies showed that the non-priority substances might have caused AhR-mediated activity. Hinger et al. [50] showed that non-priority PAHs and NSO-Het are very potent AhR agonists. Heterocyclic polyaromatic compounds, including dinaphthofurans, 2-(2-naphthalenyl) benzothiophene, methylated chrysene, and benz[a]anthracene, were identified and confirmed as major cytochrome P4501A (CYP1A)-inducing compounds in a contaminated sediment of Bitterfeld (Germany) [63]. Furthermore, Brack et al. [11] demonstrated that PAHs, especially PAHs with a molecular weight between 228 and 252 g/mol, could explain the major dioxin-like potencies of sediment extracts from the Neckar river basin.

The potential contribution of non-priority pollutants to environmental hazards was indicated in this study. To better assess the environmental samples, a broader range of substances should be considered, and studies should not only focus on prioritized pollutants. Identification of unknown pollutants that cause the main AhR-mediated activity is recommended. EDA is a suitable tool for identifying the compounds.

## Conclusion

The present study assessed the ecotoxicological hazard potential of the sediments from the Yangtze River estuary using NR retention and EROD induction assays with RTL-W1 cells. The results showed that cytotoxicity and AhR-mediated activity of sediment from Yangtze River estuary were at a minor to medium level when compared with those from other river systems. Concentrations of organic pollutants may be lower in the Yangtze River than in the Rhine River because of the comparably higher mass transport of water and particulate matter in the former, but such mass transport still results in comparably larger amounts of organic pollutants that end up in the Yangtze River’s receiving water body. At the same contamination levels in both rivers, the 14-fold amount of toxic substances would still enter the East China Sea. Therefore, the effect of the Yangtze River on the East China Sea needs to be considered.

Results of the fractionation showed that weakly and moderately polar components and polar components showed the highest AhR-mediated activities and can maximum exceed about 60% of the crude extract. A combined analysis of chemical measurements of PAHs and the results from bioassays revealed that priority EPA-PAHs contributed only a minor portion of the determined AhR-mediated activities. Furthermore, identification of unknown pollutants causing the high biological AhR-mediated potency should be the focus of future research. EDA is a suitable tool for identifying the unknown pollutants and can be used in further studies to better protect the estuary and to serve as reference for environmental monitoring in this region. Moreover, for the protection of the Yangtze River estuary, some non-priority pollutants, which cause high ecotoxicity, should also be monitored.

## Supporting Information

Table S1 **Locations for the nine samples in this study.**
(DOCX)

Table S2 **Cytotoxicity and dioxin-like activities of the crude sediment extracts in the RTL-W1 cells.** NR_50_ values for sediment extracts are given in mg sediment equivalent per mL medium (mg/mL). Dioxin-like activity expressed as biological toxicity equivalents (Bio-TEQ) in pg/g dw.
(DOCX)

Table S3 **The dioxin-like activity of the multilayer fractions in the RTL-W1 cells and expressed as biological toxicity equivalents (Bio-TEQ) in pg/g dw.**
(DOCX)
