# Supplementary material for: Evaluation of the Ecotoxicity of Sediments from Yangtze River Estuary and Contribution of Priority PAHs to Ah Receptor-Mediated Activities
Source: PLoS One. 2014 Aug 11;9(8):e104748. doi: 10.1371/journal.pone.0104748 (PMC4128779; doi:10.1371/journal.pone.0104748)
Supplement: Table S1 — Locations for the nine samples in this study. (DOCX) [file pone.0104748.s001.docx]

Table S1. Locations for the nine samples in this study

| Locations | Longitude (°) | Latitude (°) | Salinity(psu) |
| --- | --- | --- | --- |
| Y1 | 120.946 | 31.783 | 0.17 |
| Y2 | 121.441 | 31.514 | 0.17 |
| Y3 | 121.658 | 31.348 | 0.19 |
| Y4 | 121.918 | 31.146 | 2.22 |
| Y5 | 122.123 | 31.001 | 16.93 |
| Y6 | 122.376 | 30.830 | 28.00 |
| Y7 | 122.620 | 30.669 | 31.00 |
| Y8 | 122.807 | 30.456 | 34.50 |
| Y9 | 122.940 | 30.289 | 34.80 |
