# Supplementary material for: Evaluation of the Ecotoxicity of Sediments from Yangtze River Estuary and Contribution of Priority PAHs to Ah Receptor-Mediated Activities
Source: PLoS One. 2014 Aug 11;9(8):e104748. doi: 10.1371/journal.pone.0104748 (PMC4128779; doi:10.1371/journal.pone.0104748)
Supplement: Table S2 — Cytotoxicity and dioxin-like activities of the crude sediment extracts in the RTL-W1 cells. NR50 values for sediment extracts are given in mg sediment equivalent per mL medium (mg/mL). Dioxin-like activity expressed as biological toxicity equivalents (Bio-TEQ) in pg/g dw. (DOCX) [file pone.0104748.s002.docx]

**Table S2. Cytotoxicity and dioxin-like activities of the crude sediment extracts in the RTL-W1 cells.** NR_50_ values for sediment extracts are given in mg sediment equivalent per mL medium (mg/mL). Dioxin-like activity expressed as biological toxicity equivalents (Bio-TEQ) in pg/g dw.

| Sampling sites | NR assay  (NR_50_, mg/mL) | EROD assay  (Bio-TEQ, pg/g dw) |
| --- | --- | --- |
| Y1 | n.d | 110.4±21.5  323.5±4.2  38.9±5.0  182.4±75.1  268.9±40.7  105.7±23.8  103.6±10.2  188.8±20.9  317.6±41.0 |
| Y2 | n.d |  |
| Y3 | n.d |  |
| Y4 | 43.4±15.4 |  |
| Y5 | 4.1±2.2 |  |
| Y6 | 20.8±4.7 |  |
| Y7 | 38.9±17.1 |  |
| Y8 | 7.0±2.6 |  |
| Y9 | 4.9±2.3 |  |

Note: n.d. = not detectable or below the detection limit. Data are given as means of three replicates ± SD.
