# Supplementary material for: Evaluation of the Ecotoxicity of Sediments from Yangtze River Estuary and Contribution of Priority PAHs to Ah Receptor-Mediated Activities
Source: PLoS One. 2014 Aug 11;9(8):e104748. doi: 10.1371/journal.pone.0104748 (PMC4128779; doi:10.1371/journal.pone.0104748)
Supplement: Table S3 — The dioxin-like activity of the multilayer fractions in the RTL-W1 cells and expressed as biological toxicity equivalents (Bio-TEQ) in pg/g dw. (DOCX) [file pone.0104748.s003.docx]

**Table S3. The dioxin-like activity of the multilayer fractions in the RTL-W1 cells and expressed as biological toxicity equivalents (Bio-TEQ) in pg/g dw.**

| Sampling sites | Multilayer fractions (Bio-TEQ, pg/g dw) | | |
| --- | --- | --- | --- |
|  | F1 | F2 | F3 |
| Y2 | n.d. | 108.2 | 85 |
| Y4 | 3.6 | 169.8 | 122.2 |
| Y5 | 23.4 | 150.6 | 100.4 |
| Y7 | n.d. | 99.9 | 44.9 |
| Y8 | 2.7 | 157.3 | 89.0 |
| Y9 | n.d. | 153.3 | 123.7 |

Note: n.d. = not detectable or below the detection limit.
